# Single Dose of Bivalent H5 and H7 Influenza Virus-Like Particle Protects Chickens Against Highly Pathogenic H5N1 and H7N9 Avian Influenza Viruses

**DOI:** 10.3389/fvets.2021.774630

**Published:** 2021-11-11

**Authors:** Jiao Hu, Peipei Peng, Jun Li, Qi Zhang, Rumeng Li, Xiaoquan Wang, Min Gu, Zenglei Hu, Shunlin Hu, Xiaowen Liu, Xinan Jiao, Daxin Peng, Xiufan Liu

**Affiliations:** ^1^Animal Infectious Disease Laboratory, School of Veterinary Medicine, Yangzhou University, Yangzhou, China; ^2^Jiangsu Co-innovation Center for Prevention and Control of Important Animal Infectious Diseases and Zoonosis, Yangzhou University, Yangzhou, China; ^3^Key Laboratory of Prevention and Control of Biological Hazard Factors (Animal Origin) for Agri-Food Safety and Quality, Ministry of Agriculture of China (26116120), Yangzhou University, Yangzhou, China; ^4^Joint International Research Laboratory of Agriculture and Agri-Product Safety, The Ministry of Education of China, Yangzhou University, Yangzhou, China; ^5^Jiangsu Key Laboratory of Zoonosis, Yangzhou University, Yangzhou, China

**Keywords:** avian influenza virus, H5N1 subtype, H7N9 subtype, virus-like particle, baculovirus expression system, bivalent vaccine, chickens

## Abstract

Both H5N1 and H7N9 subtype avian influenza viruses cause enormous economic losses and pose considerable threats to public health. Bivalent vaccines against both two subtypes are more effective in control of H5N1 and H7N9 viruses in poultry and novel egg-independent vaccines are needed. Herein, H5 and H7 virus like particle (VLP) were generated in a baculovirus expression system and a bivalent H5+H7 VLP vaccine candidate was prepared by combining these two antigens. Single immunization of the bivalent VLP or commercial inactivated vaccines elicited effective antibody immune responses, including hemagglutination inhibition, virus neutralizing and HA-specific IgG antibodies. All vaccinated birds survived lethal challenge with highly pathogenic H5N1 and H7N9 viruses. Furthermore, the bivalent VLP significantly reduced viral shedding and virus replication in chickens, which was comparable to that observed for the commercial inactivated vaccine. However, the bivalent VLP was better than the commercial vaccine in terms of alleviating pulmonary lesions caused by H7N9 virus infection in chickens. Therefore, our study suggests that the bivalent H5+H7 VLP vaccine candidate can serve as a critical alternative for the traditional egg-based inactivated vaccines against H5N1 and H7N9 avian influenza virus infection in poultry.

## Introduction

H5N1 avian influenza virus (AIV) has been widely circulating in China and spread to more than 60 countries, and has caused huge economic losses to the poultry industry worldwide (1, 2). In addition to the serious threat to the poultry industry, H5N1 subtype AIV also raises pandemic concerns. As of September 9, 2021, H5N1 AIV has infected 863 people and caused 456 deaths (https://www.who.int/influenza/human_animal_interface/H5N1_cumulative_table_archives/en/). Other than H5N1 AIV, H7N9 AIV also has a significant impact on poultry and human health. In April 2013, three residents in Shanghai and Anhui Province, China, showed rapidly developing lower respiratory infections and were found to be infected with a new re-assortment of AIV known as low pathogenic H7N9 AIV (3). Later in 2017, highly pathogenic (HP) H7N9 AIV emerged, and caused severe human infection and disease outbreaks in poultry (4–6). As of June 3, 2021, H7N9 AIV has infected 1,568 people and caused 616 deaths (http://www.fao.org/ag/againfo/programmes/en/empres/H7N9/situation_update.html). Therefore, considering the dual threat to poultry and public health posed by H5N1 and H7N9 AIVs, a bivalent influenza vaccine is needed to prevent a potential pandemic caused by these two subtypes.

Massive vaccination of the H5+H7 trivalent (H5: Re-11; H5: Re-12; H7: Re-2) vaccine in China contributes substantially to the successful control of influenza virus outbreaks (7, 8). However, the current commercial vaccine is a whole virus inactivated vaccine and relies on embryonated chicken eggs (ECEs) for production, which has major shortfalls including insufficient supply of fertilized ECEs during influenza outbreaks, a long time period required for mass production, endogenous virus contamination, and environmental burden caused by biohazards (9, 10). Recently, bivalent inactivated vaccines based on the influenza virus vector, aiming to yield two antigens in a single virus inoculation, were generated and appeared to be immunogenic and efficacious against both HP H5N6 and H7N9 viruses in chickens (11, 12). However, production of these whole virus vaccines is still dependent on the ECEs system. Therefore, there is an urgent need to develop potential alternatives of the traditional ovoculture-based H5N1 and H7N9 influenza vaccines.

Non-infectious virus-like particle (VLP) represents one of the most promising alternatives for the traditional inactivated vaccines, owing to its ease of production and scalability, intrinsic safety, and stimulation of strong and persistent immunological response (13–16). Furthermore, VLP vaccines can provide excellent protection against homologous, heterologous, and heterosubtypic virus infections (9, 17–19), attributing to its strong ability to stimulate a comprehensive immune response, including humoral, mucosal, and cellular immunities (20–24). Numerous studies have been conducted to generate poultry VLP vaccines and the majority of these studies centered on production of H5 VLP in various expression systems, including baculovirus (25–37), silkworm pupae (38, 39), and mammalian 293T cells (40), for chicken or duck use. In addition, VLP candidates against other subtypes, such as the H6 and H7 subtypes, were also generated for chicken vaccination, including plant expression system for H6 VLP (41, 42), chicken H7 VLP generated from baculovirus (43), and silkworm pupae (44), as well as *Escherichia coli* (45). However, currently, there is limited data regarding the development of bivalent H5N1 and H7N9 VLP vaccines for chickens.

In this study, a bivalent H5+H7 VLP vaccine was prepared by combining H5 and H7 VLP assembled in a baculovirus expression system. The bivalent H5+H7 VLP vaccine provided good protection against HP H5N1 and H7N9 AIV, significantly inhibited viral shedding as well as virus replication in immunized chickens. In addition, the bivalent VLP vaccine was better than the commercial vaccine regarding protection from lung injury caused by HP H7N9 virus infection. Therefore, the bivalent H5+H7 VLP vaccine may serve as an important alternative for the egg-based inactivated vaccines for controlling these two subtypes in poultry.

## Materials and Methods

### Ethics Statement

This study was carried out in strict accordance with the recommendation in the Guide for the Care and Use of Laboratory Animals of the Ministry of Science and Technology of the People's Republic of China. The protocols for animal experiments were approved by the Jiangsu Administrative Committee for Laboratory Animals (approval number: SYXK-SU-2016-0020) and complied with the guidelines of Jiangsu Laboratory Animal Welfare and Ethics of Jiangsu Administrative Committee of Laboratory Animals. All experiments involving live viruses and animals were housed in negative-pressure isolators with HEPA filters in biosafety level 3 (BSL3) animal facilities at Yangzhou University in accordance with the institutional bio-safety manual.

### Cells and Viruses

Spodoptera frugiperda Sf9 cells (ATCC # CRL-1711) were maintained in SF900III insect serum-free medium (SFM) (ThermoFisher Scientific, Rockford, IL) supplemented with 5% fetal calf serum (FCS) (Invitrogen, CA) at 27°C. Sf9 suspension cells were routinely cultured in insect serum-free SF900II SFM (ThermoFisher Scientific) at 27°C in spinner flasks at a speed of 120 rpm. Chicken embryo fibroblasts (CEF) were cultured in M199 medium supplemented with 5% FCS and maintained at 37°C with 5% CO_2_. HP H7N9 virus A/chicken/Guangdong/GD15/2016 (GD15) and H5N1 virus A/chicken/Shandong/TT3/2016 (TT3) were isolated and identified previously (46). The viruses were plaque-purified for three rounds in CEF cells and propagated in 10-day-old specific-pathogen-free (SPF) ECEs (Beijing Merial Vital Laboratory Animal Technology Co., Ltd., Beijing, China). The GD15 virus was used as H7 HA, NA, and M1 gene donor, while the TT3 virus was used as H5 HA gene donor. These two viruses were also used as a homologous challenge virus in chicken immunization studies.

### Generation of the Recombinant Baculoviruses

Four recombinant pVL1393 transfer plasmids encoding GD15 HA (pVL1393-H7), TT3 HA (pVL1393-H5), GD15 NA (pVL1393-NA), or GD15 M1 (pVL1393-M1) gene was generated as described previously (47). To generate the recombinant baculoviruses (rBVs), Sf9 insect cells were transfected with 500 ng of each plasmid and 100 ng of the linearized genomic DNA of *Autographa californica multiple nucleopolyhedro virus* (AcMNPV). After 72 h post-transfection, indirect immunofluorescence assay (IFA) was carried out to verify the rescue of these rBVs. The rescued rBVs were then plaque-purified for three rounds in Sf9 cells and were serially propagated in Sf9 cell suspension culture for several passages. The rBVs were designated as rBac-H7, rBac-H5, rBac-NA, and rBac-M1, respectively.

### Indirect Immunofluorescence Assay

To detect viral protein expression by the rBVs, Sf9 cells were infected with the rBVs at a multiplicity of infection (MOI) of 1. At Day 4 post-infection (p.i.), the cells were processed for IFA as described previously (47). Briefly, the cells were washed and fixed with cold methanol for 20 min at 4°C. The fixed cells were then incubated with the primary antibodies, including mouse monoclonal antibody (mAb) against the H7N9 HA protein (Sino Biological, Beijing, China), rabbit polyclonal antibody against the H5N1 HA protein (Sino Biological), rabbit mAbs against the H7N9 NA (GeneTex, Irvine, CA) or M1 proteins (Bioss, Beijing, China) for 1 h at 37°C, respectively. After washing with PBS, the secondary antibodies, Alexa Fluor 488 conjugated goat anti-rabbit IgG (H+L) or Alexa Fluor 594 conjugated goat anti-mouse IgG (H+L) (Sino Biological), were added to the cells and incubated at 37°C for 1 h. Fluorescence signal was observed under a Leica fluorescence microscope. To determine 50% tissue culture infectious dose (TCID_50_) of the rBVs, serial dilutions of the indicated rBVs were added to Sf9 cells in 96-well plates. After 96 h p.i., baculovirus infection was determined by detecting expression of baculovirus envelope gp64 protein using the mouse mAb against gp64 (eBioscience, CA) with IFA.

### Preparation of the Bivalent H5 and H7 VLP Vaccine

H5 and H7 VLP was assembled in Sf9 cells individually and combined to prepare the bivalent H5+H7 VLP. To generate the H7 VLP, Sf9 suspension cells (2 × 10^6^ cells/mL) were co-infected with the rBac-H7, rBac-NA, and rBac-M1 at a MOI ratio of 1:1:1. The H5 VLP was produced according to the same procedure. Culture supernatants were harvested at Day 3 p.i. and clarified by low-speed centrifugation (2,000 *g* for 20 min at 4°C) followed by ultracentrifugation at 10,000 *g* for 2 h at 4°C. The obtained VLP suspension was then placed on formvar-coated (copper 300 mash) grids, negatively stained with 1% uranyl acetate, and dried by aspiration. The VLP particles were then examined under a transmission electron microscope as described previously (17). For further purification, the VLP was concentrated by ultrafiltration and the pellet was then resuspended in PBS and stored at 4°C. A BCA protein assay kit (Beyotime, Nantong, China) was used to determine protein concentration of the H5 and H7 VLP. For preparation of the bivalent H5 and H7 VLP vaccine candidate, 7.5 μg of each VLP antigen was mixed and adjuvanted with MONTANIDETM ISA 71R VG at a volume ratio of 1:2 (Seppic, Paris, France).

### Immunization and Challenge Studies in Chickens

To investigate the immunogenicity and efficacy of the bivalent H5+H7 VLP in chickens, 64 4-week-old SPF White Leghorn chickens (*Gallus gallus domesticus*) (Beijing Experimental Animal Center, Beijing, China) were divided into two group (*n* = 32). Birds were intramuscularly (i.m.) immunized with 15 μg of the bivalent H5+ H7 VLP, or 0.3 mL of the commercial trivalent (H5: Re-11; H5: Re-12; H7: Re-3) vaccine (Yebio, Qingdao, China) according to the label. At Week 3 post-vaccination (p.v.), the vaccinated birds of each group were randomized into two subgroups and moved into the biosafety level-3 animal facility for virus challenge. One group (*n* = 16) was challenged with 10^6.0^ 50% embryo infectious dose (EID_50_) (in 100 μL) of the H5N1 TT3 virus, another group (*n* = 16) was challenged with 10^6.0^ EID_50_ the HP H7N9 GD15 virus.

Moreover, another group of 22 birds was inoculated with 0.3 mL of PBS as the un-immunized control. At Week 3 p.v., birds were randomized into two subgroups and moved into the biosafety level-3 animal facility for virus challenge. One group (*n* = 11) was challenged with the highly pathogenic H5N1 TT3 virus, another group (*n* = 11) was challenged with the highly pathogenic H7N9 GD15 virus.

Sera samples were collected at Week 2 and 3 p.v. The infected birds were then observed twice a day for clinical signs for 14 days post-challenge (p.c.). The mortality was recorded daily. Those severely ill or moribund birds were euthanized by inhalation of carbon dioxide and counted in mortality the following day. To monitor viral shedding, oropharyngeal and cloacal swabs were collected on Day 2, 4, and 6 p.c. for virus isolation in ECEs. To determine viral replication in birds, on Day 2 and 4 p.c., three birds of each group were euthanized, and the heart, cecum, lung, and spleen were collected for virus titration in ECEs. The remaining birds of each group were observed for clinical symptoms and mortality.

### Histological Examination

To evaluate the protective effect of vaccination on the lung injury caused by virus infection, the chicken lung from each group (*n* = 3) was collected on Day 2 and 4 p.c. and fixed with 10% neutral formaldehyde and embedded in paraffin. Tissue sections were then cut 5 μm thick and stained by hematoxylin and eosin (H & E). Histological changes were then examined by a double-blind method and captured with a light microscope (Olympus, Japan) as described previously (48). Lung lesions were scored according to the following standards: 0, no visible changes; 1, mild lesions, including dilation and minor congestion in parabrochus and/or pulmonary chamber; a small amount of detached epithelial cell mucus in the parabrochus; 2, moderate lesions, including moderate dilation, congestion or hemorrhage in parabrochus and/or pulmonary chamber; abundant detached epithelial cell mucus in the parabrochus; a few lymphocytes infiltration in blood vessels, parabrochus, and/or pulmonary chamber; 3, severe lesions, including severe dilation, congestion, or hemorrhage in bronchia, parabrochus, and/or pulmonary chamber; extensive lymphocytes infiltration around the parabrochus and/or pulmonary chambers; a large amount of detached epithelial and lymphatic mucus in the bronchial and parabronchial lumen.

### Hemagglutination Inhibition Assay

Hemagglutination inhibition (HI) assay was performed using 1% chicken erythrocytes with 4 HA unit (HAU) of the homologous viruses using the standard method (49). Briefly, the chicken sera were serially diluted and incubated with 4 HAU of H7N9 GD15 strain or H5N1 TT3 virus at 37°C for 10 min, and then incubated with 25 μL of 1% chicken erythrocytes. Plates were read after 30 min of incubation at room temperature. HI titers were recorded as the highest serum dilution that completely inhibited the hemagglutination.

### Virus Neutralization Assay

Virus neutralizing (VN) antibody titers of the serum samples were determined as described previously (50). Briefly, a monolayer of CEF cells were cultured in M199 medium supplemented with 5% FCS. Heat-inactivated sera were serially diluted with M199 medium containing 1% FCS and mixed with equal volume of H7N9 GD15 virus (100 TCID_50_) or H5N1 TT3 virus (100 TCID_50_). After incubation at 37°C for 1 h, the virus-sera mixture was then transferred to CEF cells in 96-well tissue culture plates and cultured for 4 days. VN antibody titers were determined as the reciprocal of the highest serum dilution that completely inhibited the cytopathic effect (CPE) caused by virus infection.

### IgG Antibody Titers Measured by ELISA

HA-specific IgG antibody titers in the sera collected at Week 3 p.v. were determined by enzyme-linked immunosorbent assay (ELISA) as described previously (51). In brief, the flat-bottomed 96-well microplate plates were coated with 250 ng of the purified HA protein of H7N9 A/Anhui/1/2013 (AH13) virus (Sino Biological) or the purified TT3 HA protein expressed in Sf9 insect cells at 4°C overnight. PBST (PBS containing 0.05% Tween 20) supplemented with 1% bovine serum albumin (BSA) were added for blocking at 37°C for 2 h. The chicken sera were serially diluted, and then added to the well and incubated at 37°C for 1 h. Then, the plates were washed with PBST three times and incubated with 100 μL of HRP-conjugated secondary IgG (Sigma, St. Louis, USA) at 37°C for 1 h. The plates were then washed with PBST three times and incubated with 100 μL of 3, 3', 5, 5'-tetramethylbenzidine (TMB) liquid substrate. The reaction was stopped by the addition 50 μL of 2M H_2_SO_4_ and the optical density (OD) was read at 450 nm using a spectrophotometer.

### Statistical Analysis

Statistical analyses were performed by the unpaired t tests using GraphPad Prism (GraphPad Software, San Diego, CA). Data are expressed as the mean ± standard deviation (SD). Statistical significance was designated for differences with *p*-values < 0.05 (^*^p < 0.05, ^**^p < 0.01, ^***^p < 0.001, ^****^p < 0.0001).

## Results

### Generation of the Recombinant Baculoviruses and Assembly of the H5 and H7 VLP

**Figure 1:**
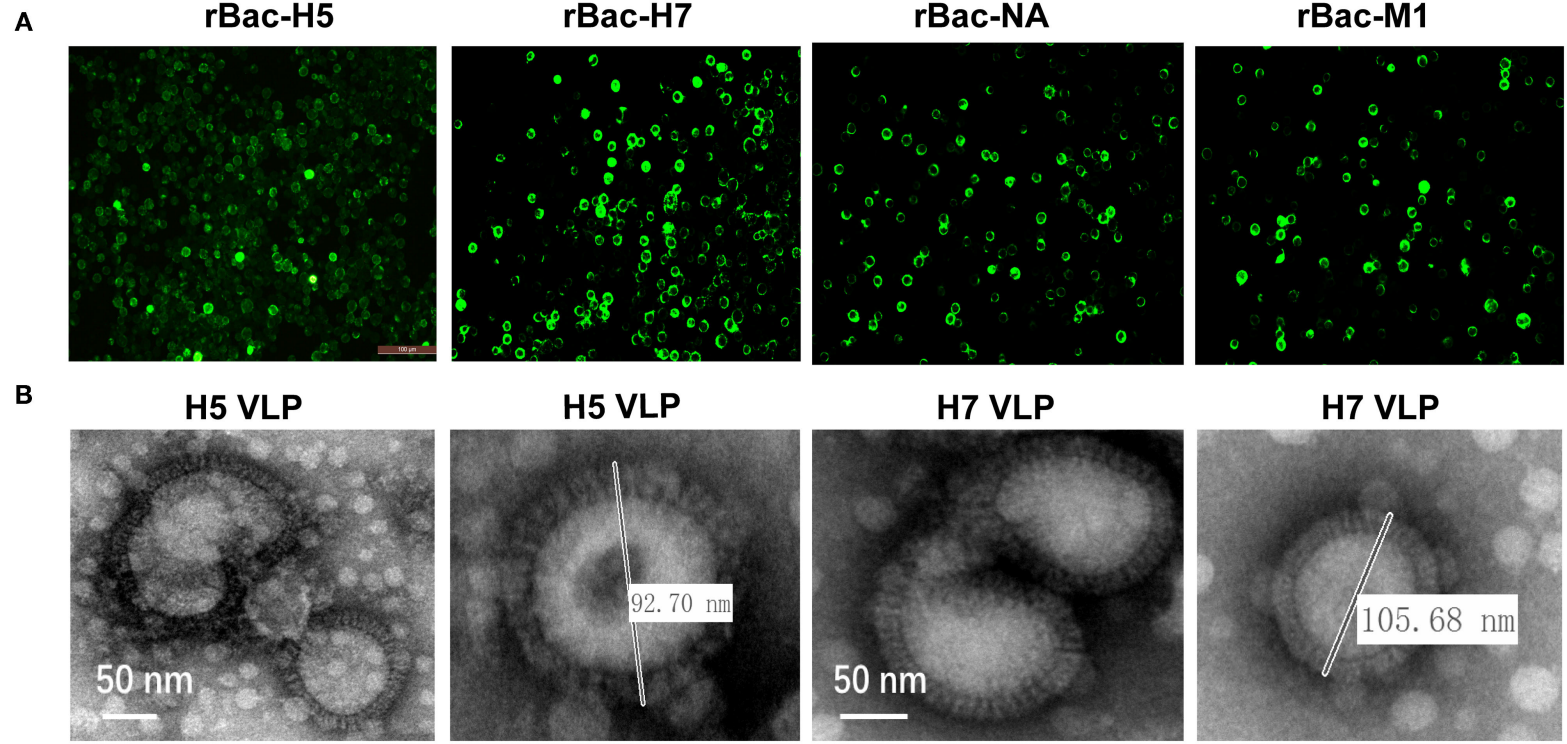
Generation and characterization of H5 and H7 VLP. **(A)** Recombinant baculovirus (rBVs) rBac-H5, rBac-H7, rBac-NA, and rBac-M1 were successfully identified using immunofluorescence assay (IFA) by HA, NA, and M1-specific antibody in Sf9 cells. **(B)** To generate H5 VLP, Sf9 suspension cells were co-infected with rBac-H5, rBac-NA, and rBac-M1 at a MOI ratio of 1:1:1. To generate H7 VLP, Sf9 suspension cells were co-infected with rBac-H7, rBac-NA, and rBac-M1 at the same MOI ratio. At 72 h p.i., cell supernatant was harvested for collection of the H5 and H7 VLP. A transmission electron microscope (EM) was used for observation of the morphology and size of the H5 and H7 VLP.

The HA genes of the H5N1 or H7N9 subtypes and the NA and M1 genes of the H7N9 subtype were amplified and ligated into the transfer plasmid pVL1393. The recombinant baculoviruses expressing the HA, NA, and M1 genes were generated through co-transfection of the transfer vectors with the linear genomic DNA of AcMNPV. The expression of the HA, NA, and M1 proteins were detected in the transfected Sf9 cells using IFA, indicating successful rescue of the recombinant baculoviruses (Figure 1A). Subsequently, the H5 and H7 VLP were assembled in Sf9 cells by co-infection of the baculoviruses expressing the HA, NA, and M1 genes. Enveloped spherical particles were observed by transmission electron microscopy and the diameters of the particles were like that of natural influenza virus particles (Figure 1B). These results showed that the H5 and H7 VLP were successfully assembled through baculovirus co-infection, which had similar morphology and size with the natural influenza virus particles.

### HI and VN Antibodies Elicited by the Bivalent H5+H7 VLP in Chickens

**Figure 2:**
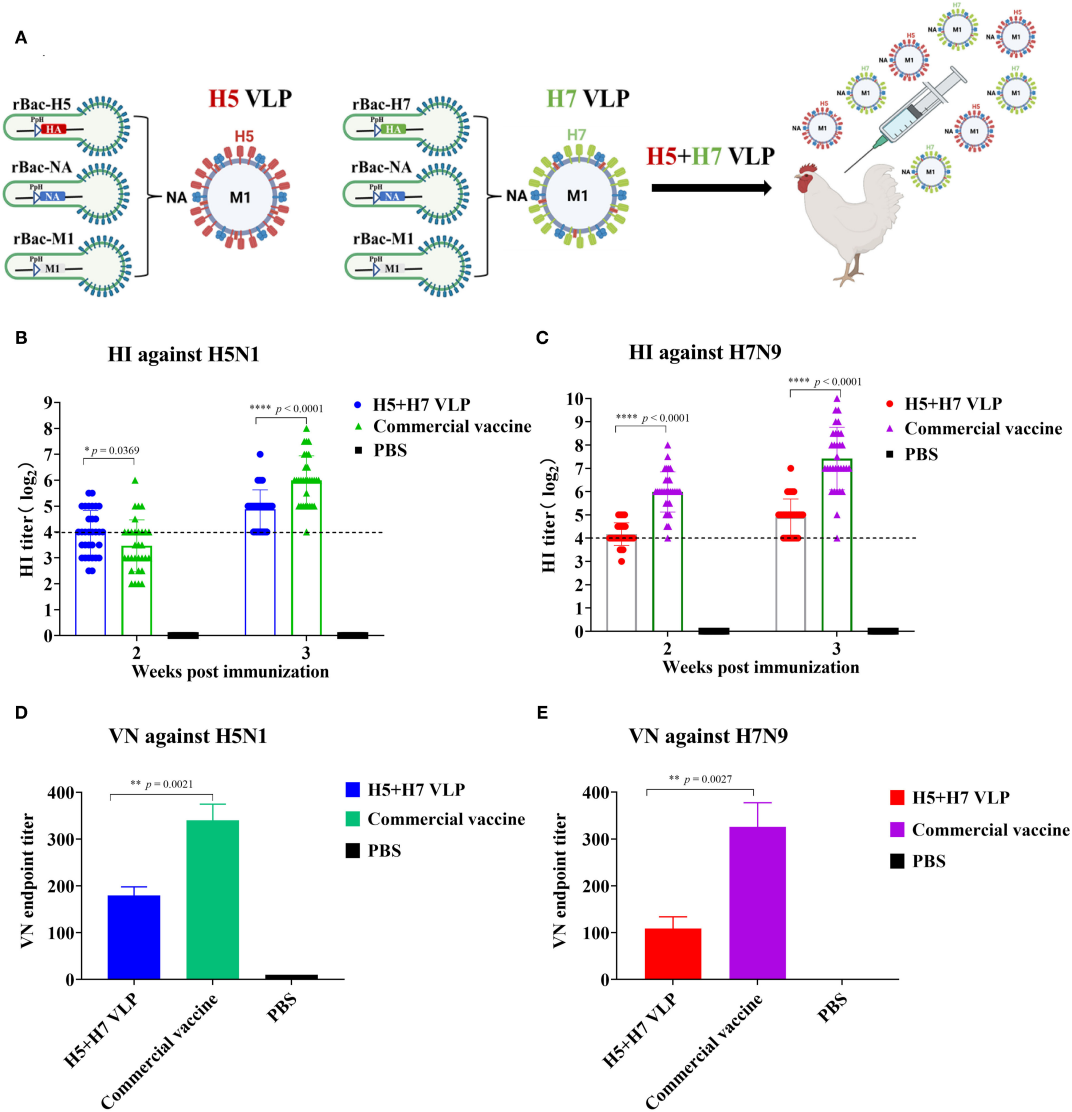
The bivalent H5+H7 VLP vaccine induces efficient HI and VN antibodies. **(A)** To assess the immunogenicity of the vaccine candidate, chickens were intramuscularly (i.m.) immunized with 15 μg of the bivalent H5+H7 VLP or 0.3 mL of the commercial trivalent (H5: Re-11; H5: Re-12; H7: Re-3) vaccine. **(B)** HI titers at Week 2 and 3 p.v. against H5N1 TT3 virus. **(C)** HI titers at Week 2 and 3 p.v. against H7N9 GD15 virus. **(D)** VN antibody against 100 TCID_50_ of H5N1 TT3 virus. **(E)** VN antibody against 100 TCID_50_ of H7N9 GD15 virus. The detection limit was below 4 log_2_ for the HI assay and below 10 for the VN assay.

The H5 and H7 VLP were purified and combined at a ratio of 1:1 to prepare the bivalent H5+H7 VLP vaccine (Figure 2A). The inclusion level of both the H5 and H7 VLP per dose was 7.5 μg. Chickens were immunized with the bivalent H5+H7 VLP and commercial inactivated vaccine, respectively. The results demonstrated that the mean HI titer against H5 induced by the VLP vaccine was 4 log_2_, significantly higher than that induced by the commercial vaccine at Week 2 p.v. (Figure 2B). However, HI antibody titers against H5 elicited by the commercial vaccine increased rapidly to 6 log_2_ at Week 3 p.v., which were significantly higher than that induced by the bivalent VLP vaccine (5 log_2_). In addition, a different profile was seen for H7-specific HI antibody response (Figure 2C). Significantly higher HI titers were detected in the chickens immunized with the commercial vaccine at Week 2 and 3 p.v. compared to the bivalent VLP vaccine. VN activity of the antisera collected at Week 3 p.v. was determined and we found that the commercial inactivated vaccine induced a significantly stronger VN antibody response against H5 and H7 in chickens (Figures 2D,E). Taken together, these findings suggest that: (1) the bivalent VLP could induce effective antibody response against H5 and H7 in chickens; and (2) HI and VN titers elicited by the VLP vaccine were significantly lower than that induced by the commercial vaccine before the virus challenge.

### IgG Antibodies Induced by the Bivalent H5+H7 VLP in Chickens

**Figure 3:**
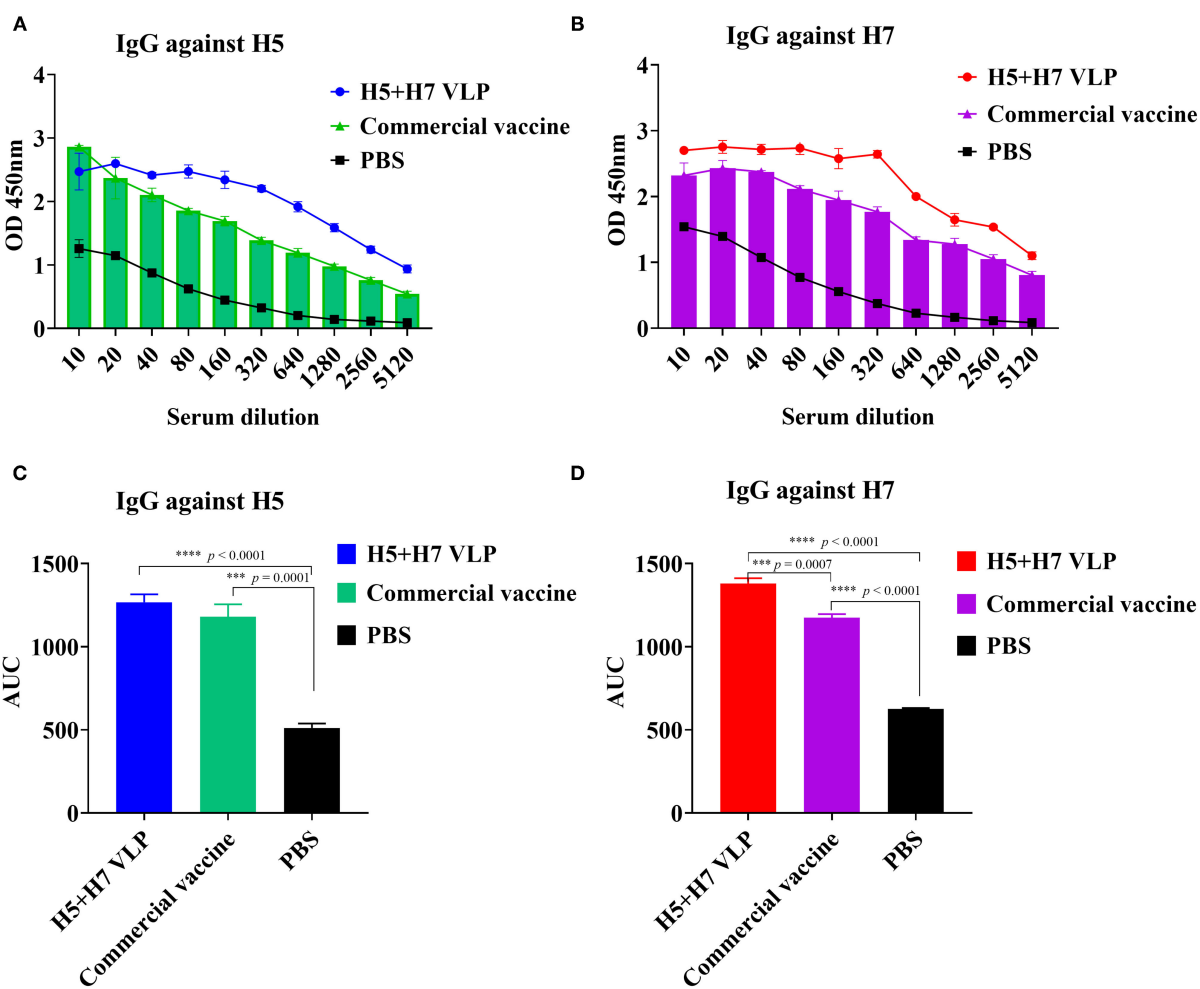
The bivalent H5+H7 VLP vaccine stimulates strong IgG antibody. Chickens were i.m. immunized with 15 μg of the bivalent H5+H7 VLP or 0.3 mL of the commercial trivalent (H5: Re-11; H5: Re-12; H7: Re-3) vaccine. The IgG antibody at Week 3 p.v. was determined by ELISA. **(A)** IgG antibody using purified HA protein of the TT3 virus as coated antigen. **(B)** IgG antibody using purified HA protein of the A/Chicken/Anhui/AH13/2013 (AH13) virus as coated antigen. **(C)** Area under the curve (AUC) of the IgG antibody using purified HA protein of the TT3 virus as coated antigen. **(D)** AUC of the IgG antibody using purified HA protein of the AH13 virus as coated antigen.

To further compare the immunogenicity of the bivalent VLP and commercial vaccine, the level of HA-binding IgG antibody was also measured using ELISA. The results revealed that both the bivalent VLP and commercial vaccine elicited high levels of H5-specific IgG antibody in chickens and antibody titers were comparable between them, although lower HI and VN titers were detected for the VLP vaccine (Figures 3A,C). Robust H7-specific IgG antibody responses were observed in chickens immunized with the VLP and commercial vaccine, while the magnitude of the IgG antibody response induced by the VLP vaccine was significantly higher than that of the commercial vaccine (Figures 3B,D). Altogether, these findings suggest that: (1) compared with the commercial vaccine, the bivalent VLP induced significantly higher HA-binding IgG antibody against H7 in chickens; and (2) the bivalent VLP and commercial vaccine showed different IgG profiles against H5 and H7.

### Protective Efficacy of the Bivalent H5+H7 VLP in Chickens

**Figure 4:**
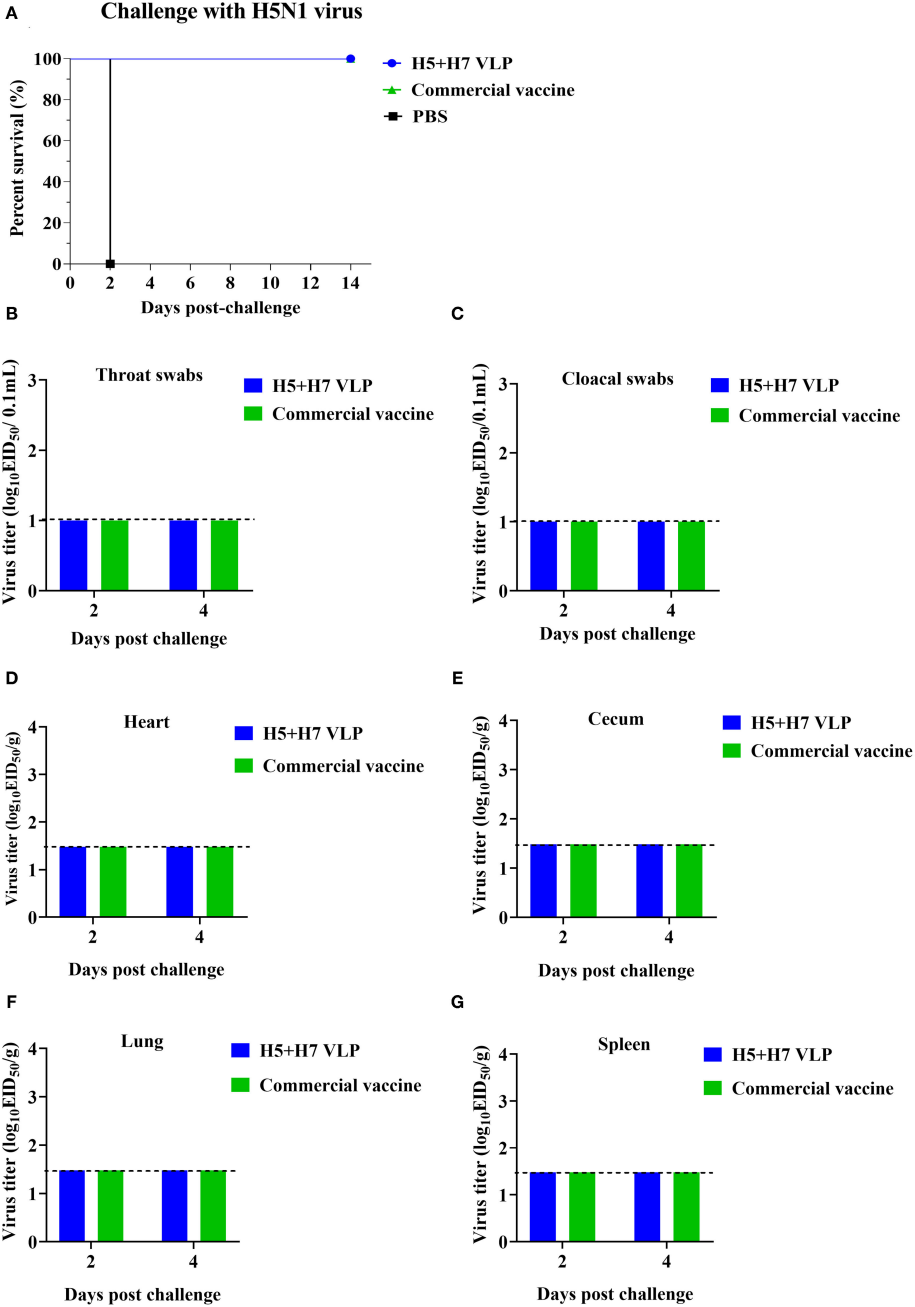
The bivalent H5+H7 VLP vaccine confers good protection against the lethal H5N1 virus. Chickens were i.m. immunized with 15 μg of the bivalent H5+H7 VLP or 0.3 mL of the commercial trivalent (H5: Re-11; H5: Re-12; H7: Re-3) vaccine. **(A)** Survival rates of the birds after challenged with 10^6.0^ EID_50_ of the H5N1 TT3 virus. **(B)** Virus titration in laryngotracheal swabs on Day 2 and 4 p.c. **(C)** Virus titration in cloacal swabs on Day 2 and 4 p.c. Viral titers in the heart **(D)**, cecum **(E)**, lung **(F)**, and spleen **(G)** of the birds on Day 3 and 5 p.c. Viral titers were determined by measuring EID_50_. The detection limit was below 10^1.0^ EID_50_/0.1 mL for swabs and 10^1.48^ EID_50_/g for organs.

**Figure 5:**
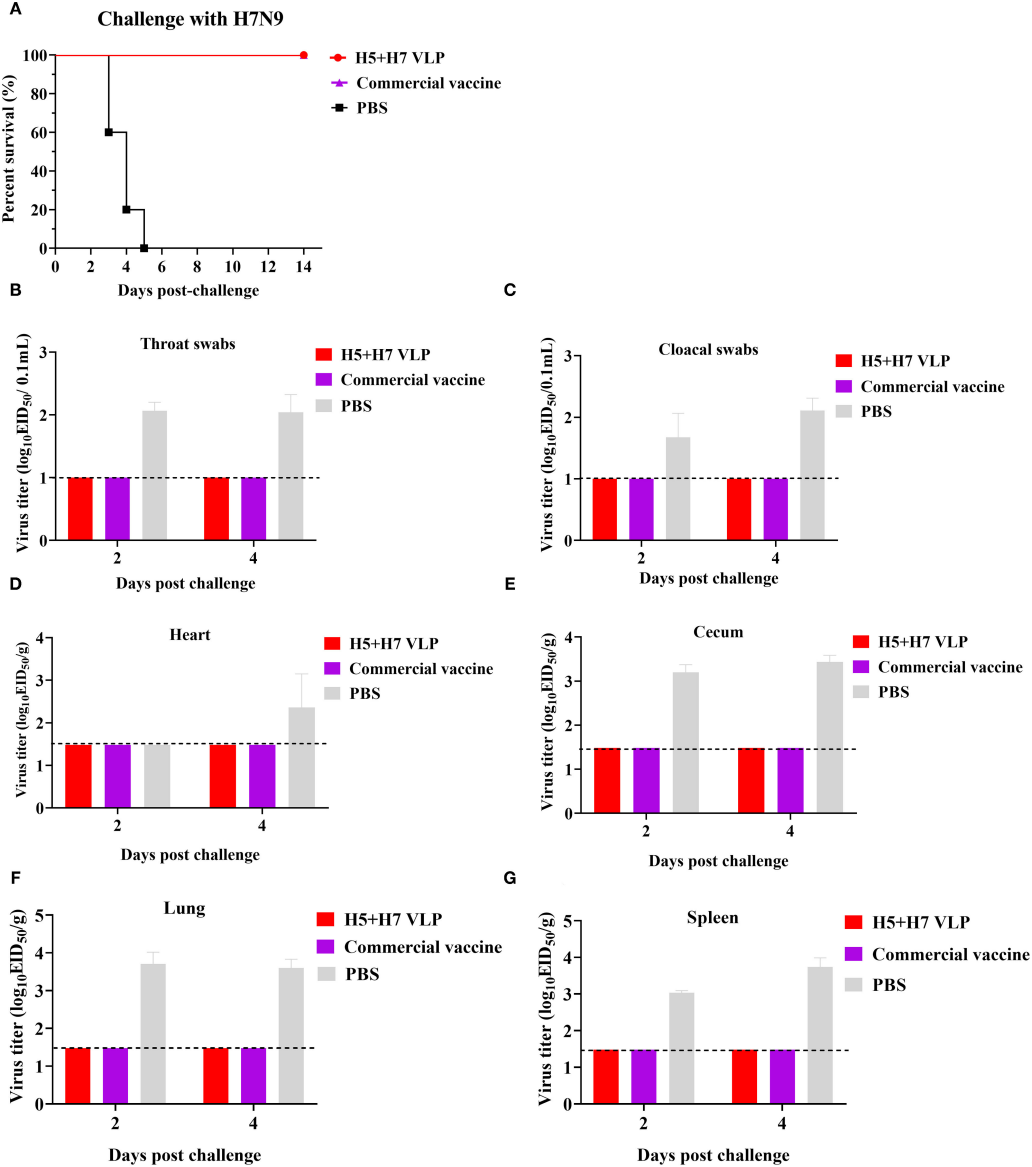
The bivalent H5+H7 VLP vaccine confers good protection against the lethal H7N9 virus. Chickens were i.m. immunized with 15 μg of the bivalent H5+H7 VLP or 0.3 mL of the commercial trivalent (H5: Re-11; H5: Re-12; H7: Re-3) vaccine. **(A)** Survival rates of the birds after challenged with 10^6.0^ EID_50_ of the H7N9 GD15 virus. **(B)** Virus titration in laryngotracheal swabs on Day 2 and 4 p.c. **(C)** Virus titration in cloacal swabs on Day 2 and 4 p.c. Viral titers in the heart **(D)**, cecum **(E)**, lung **(F)**, and spleen **(G)** of the birds on Day 3 and 5 p.c. Viral titers were determined by measuring EID_50_. The detection limit was below 10^1.0^ EID_50_/0.1mL for swabs and 10^1.48^ EID_50_/g for organs.

To evaluate the protective efficacy of the vaccine, each group of the bivalent VLP or the commercial vaccine immunized chickens were randomized into two subgroups (*n* = 16) and challenged with AIV of the H5 or H7 subtypes, respectively. After lethal virus challenge, the unvaccinated chickens rapidly died with typical clinical signs caused by HP influenza virus infection, e.g., skin cyanosis, depression, facial edema, hemorrhage of foot scale, and central nervous system (CNS) signs. In contrast, all the vaccinated chickens survived, without showing any clinical signs during a 14-day observation period (Figures 4A, 5A). Throat and cloacal swabs were collected at Day 2 and 4 p.c. to monitor virus shedding. No swabs were taken from the mock chickens challenged with H5N1 virus because they all died at the sampling time (Figure 4A). All the mock-vaccinated chickens challenged by H7N9 virus shed virus (Table 1), with titers around 10^2^ TCID_50_/0.1mL via the oropharynx or the cloaca at Day 2 and 4 p.c. (Figures 5B,C). In contrast, none of the vaccinated birds challenged by H5N1 (Figures 4B,C) or H7N9 virus (Figures 4C, 5B) shed virus during the entire observation period. Additionally, the tissues were also collected to assess the inhibitory effect of vaccination on systematic dissemination of the challenge viruses. The unvaccinated chickens died within 2 days after H5 virus challenge, suggesting the occurrence of a highly lethal systematic infection, despite that no tissue samples were collected. In the unvaccinated chickens after H7 virus challenge, virus was detected in the heart from 2 out of 3 birds at Day 4 p.c., and in the cecum, lung, and spleen from all three birds at Day 2 and 4 p.c., indicating a systematic infection of the virus (Figures 5D–G). By contrast, no virus was detected in the tissues collected from the vaccinated birds at Day 2 and 4 p.c when challenged with HP H5N1 (Figures 4D–G) or H7N9 virus (Figures 5D–G). Collectively, these results indicate that both the bivalent H5+H7 VLP and commercial vaccine can protect chickens from clinical disease and mortality and inhibit virus shedding and replication which are associated with HP H5N1 and H7N9 virus infection.

**Table 1:** Virus shedding of the birds challenged by H5N1 or H7N9 virus.

**Groups^d^**	**Challenge virus**	**2 dpi^a^**		**4 dpi**		**6 dpi**		**Virus shedding/total**	**Survival/total**
		**L^b^**	**C^c^**	**L C**	**L C**	**L C**	**L C**		
H5+H7 VLP	H5N1/TT3	0/10	0/10	0/10	0/10	0/10	0/10	0/10	10/10
Commercial vaccine	H5N1/TT3	0/10	0/10	0/10	0/10	0/10	0/10	0/10	10/10
H5+H7 VLP	H7N9/GD15	0/10	0/10	0/10	0/10	0/10	0/10	0/10	10/10
Commercial vaccine	H7N9/GD15	0/10	0/10	0/10	0/10	0/10	0/10	0/10	10/10
PBS	H5N1/TT3	-^d^	-	-	-	-	-	-	0/5
PBS	H7N9/GD15	4/5	3/5	1/1	1/1	-	-	5/5	0/5

a *dpi, days post infection*.

b *L, Laryngotracheal swabs*.

c *C, Cloacal swabs*.

d *-, samples were not collected since birds were all dead*.

### Protection Against Lung Pathological Changes Conferred by VLP Vaccination

**Figure 6:**
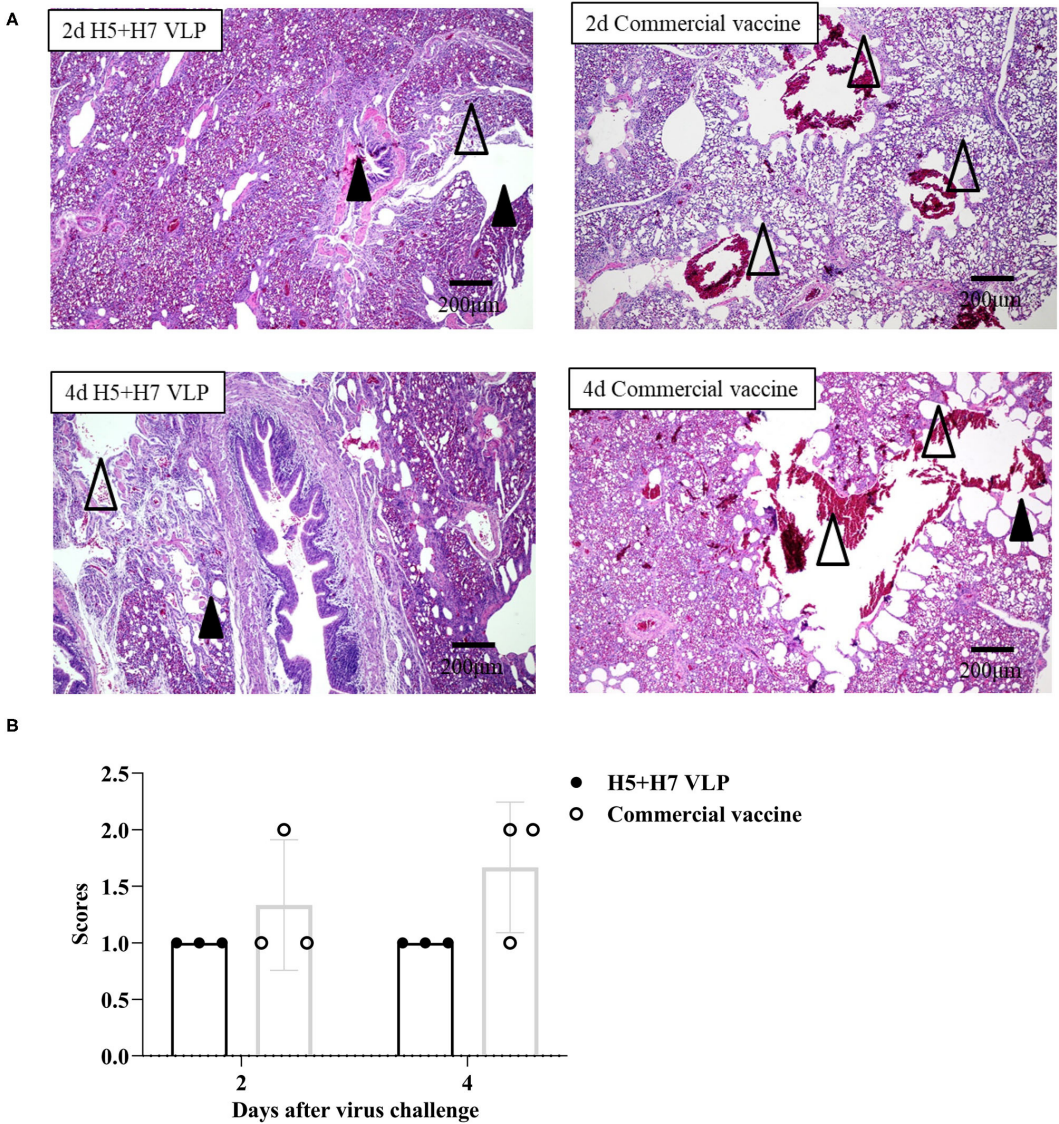
The bivalent H5+H7 VLP vaccine inhibits lung pathological changes after the lethal H5N1 virus challenge. **(A)** Hematoxylin and eosin (H & E) staining results of the birds' lung on Day 2 and 4 post-H5N1 virus challenge. Black triangle indicates dilatation in the pulmonary chamber or bronchial; white triangle arrow stands for infiltration of fiber cells, detached epithelial cell mucus, and erythrocytes in the parabrochus. **(B)** Scores of the overall histopathologic changes in the bird's lung post-H5N1 virus challenge. The specific information concerning the scoring criteria was listed in the Materials and Method section.

**Figure 7:**
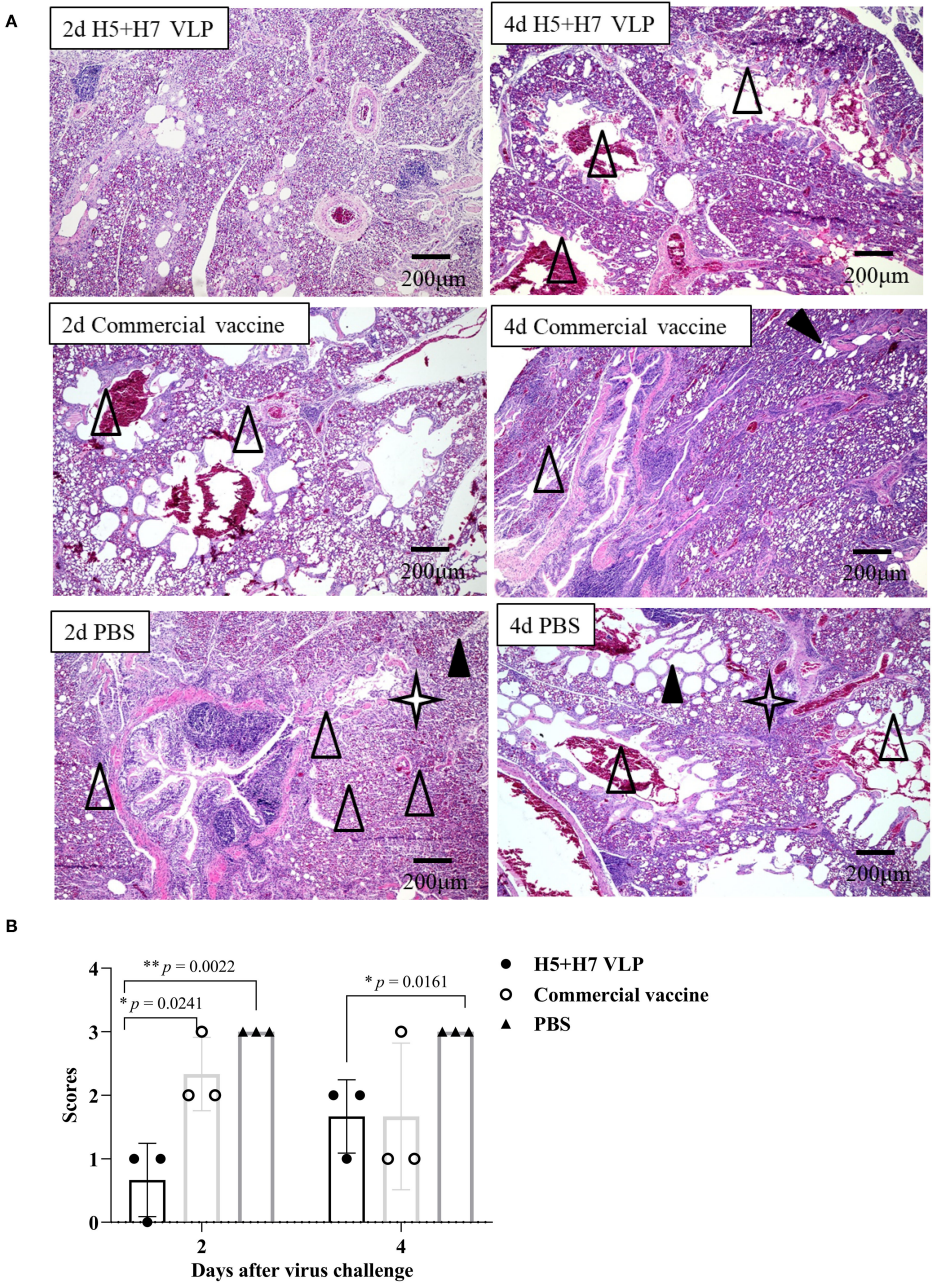
The bivalent H5+H7 VLP vaccine alleviates lung injury after the lethal H7N9 virus challenge. **(A)** H & E staining results of the bird's lung on Day 2 and 4 post-H7N9 virus challenge. Black triangle indicates dilatation in the pulmonary chamber or bronchial; white triangle arrow stands for infiltration of fiber cells, detached epithelial cell mucus, and erythrocytes in the parabrochus; quad star means lymphocytes infiltration in the lung chamber. **(B)** Scores of the overall histopathologic changes in the bird's lung post-H7N9 virus challenge. The specific information concerning the scoring criteria was listed in the Materials and Method section.

Suppression of pathological lesions caused by virus infection is a critical parameter of vaccine efficacy. Histopathological changes in the lung of three chickens at Day 2 and 4 p.c. challenged with H5 and H7 AIV were assessed. Lung section of the mock control birds were not collected since all the birds died rapidly after challenged with the HP H5N1 TT3 virus. As for the vaccinated birds, when the birds were challenged with the H5N1 TT3 virus, the lung of the bivalent VLP vaccinated chickens all showed mild histological changes at Day 2 and 4 p.c., including mild dilatation in the pulmonary chamber or bronchial and infiltration of fiber cells, few detached epithelial cell mucus and erythrocytes in parabrochus (Figure 6A). By contrast, some of the commercial vaccine immunized birds showed severe histological changes in the lung at Day 2 and 4 p.c., while no significant differences were observed between the two vaccine groups (Figure 6B). When comparing the histological changes caused by HP H7N9 virus infection, we found that only slight lung injury was observed in VLP vaccination groups, resulting in significantly lower extent lung injury than that of the unimmunized birds (Figure 7). In contrast, the commercial vaccine failed to alleviate the lung pathology caused by the HP H7N9 virus infection in some of the vaccinated birds. To be noted, at Day 2 p.c., when challenged with H7N9 virus, the birds immunized with the commercial vaccine showed significantly severe lung injury than that of the VLP group (Figure 7B). Therefore, these results demonstrated that the VLP was better than the commercial vaccine in terms of alleviating pulmonary lesions caused by H7N9 virus infection in chickens.

## Discussion

H5N1 and H7N9 AIV are enzootic in domestic poultry in China (8, 52–54). Rational use of vaccination can be an important measure for prevention and control of these two subtypes. To contain infection and spread of H5N1 and H7N9 viruses, a trivalent inactivated commercial H5+H7 vaccine has been administered in poultry flocks throughout China, resulting in a remarkable decrease in virus dissemination (7, 8). However, the current commercial vaccine is manufactured by the traditional egg-based approach and has some drawbacks. Therefore, there is a great need to develop a new modality for avian influenza vaccine. In this study, the H5 and H7 VLP was generated in a baculovirus expression system and a bivalent H5+H7 vaccine was prepared by combining these two antigens. The bivalent VLP was immunogenic against H5N1 and H7N9 viruses in chickens, while it induced lower HI and VN antibody titers than the commercial inactivated vaccine (Figure 2). Both the bivalent VLP and commercial vaccine provided good protection against challenge with H5N1 and H7N9 viruses and significantly suppressed virus shedding and infection. Of note, the bivalent VLP vaccine displayed a greater capacity of inhibiting pathological changes in the lung caused by H7N9 virus (Figure 7). Therefore, the bivalent VLP may be used as an important alternative to the traditional egg-based vaccines for control of H5N1 and H7N9 AIV in poultry.

Broadly protective vaccines based on multivalence strategy have been widely used in seasonal influenza, pneumococcal vaccines, and human papillomavirus (55). Combination of H5 and H7 antigens to produce bivalent vaccines is a common practice for preparation of traditional inactivated vaccine in poultry. However, there is a concern about this approach that propagation of individual H5 and H7 viruses needs more ECEs, leading to increase of cost-of-good and yield of biohazards. Aiming to yield two antigens by a single virus inoculation, some studies were performed to generate recombinant influenza viruses expressing the HA proteins from two subtypes. Li et al. generated a reassortant H5N6 virus based on PR8 backbone expressing the H7N9 HA1 in-frame in the NS1 gene (11). The reassortant virus was highly immunogenic and efficacious against both the H5 and H7N9 HPAIV in chickens, however, no comparison with the commercial vaccine was done. Nevertheless, production of bivalent vaccines based on these viruses relies on an egg-dependent system. It is accepted that the traditional egg-based approach for influenza vaccine production has drawbacks, such as a shortage of egg supply, yield of large amounts of biohazards, lack of mucosal and cellular immunity, and potential risk of endogenous virus contamination (9, 10, 56, 57).

The VLP system is a promising platform for development of novel vaccines as alternatives for inactivated vaccines (58–60). Numerous vaccine candidates against different influenza subtypes were generated using the VLP platform (61). However, reports on the development of avian bivalent or multivalent influenza vaccines are limited. In this study, using the established VLP platform, we generated the H5 and H7 VLPs in Sf9 insect cells and made a bivalent VLP vaccine through antigen combination strategy. The individual VLP antigen was prepared by co-infection with three baculoviruses expressing the HA, NA, and M1 genes, respectively, which are required for influenza VLP assembly (Figure 1). A previous study used a different strategy for generation of a trivalent VLP vaccine displaying H5, H7, and H9 HA (32). Insect cells were infected with the recombinant baculovirus co-expressing the retroviral gag, N1 and HA proteins of H5, H7, and H9 for VLP assembly. Of note, the efficiency of the VLP assembly through the co-infection and co-expression strategies needs to be compared. Furthermore, a two-dose regimen was used in an efficacy experiment in that study, while herein we showed that a single dose of the VLP vaccine provided good protection from clinical signs, virus shedding, and systematic infection post-challenge with the homologous wild type strain. However, further studies are needed to determine whether a single dose of the bivalent VLP vaccine could confer full protection against challenge with a heterologous strain.

Recently, a novel bioreactor, silkworm pupae, was also used for production of bivalent H5+H7 influenza VLP vaccines through co-infection with recombinant baculoviruses (62). However, in that study, pupae were infected with only two baculoviruses expressing the H5 and H7 HA genes, and thus the obtained VLP might be the subviral particles formed by the HA protein because no scaffold protein, such as the M1 and gag, for VLP assembly, was supplied. Such subviral particles formed by the H7 HA protein alone were also reported previously (63). The major advantage of a pupae system is the high antigen yield, evidenced by around 300,000–500,000 HAU for H5 and H7 component in 20 pupae, respectively. Both insect cells and silkworm bioreactor are promising systems for large-scale VLP production (23, 64–66), and antigen yield, efficiency of VLP assembly, antigen process, and purification of these two systems should be compared systematically in the future.

Another significance of the present study was the comprehensive comparison between the bivalent VLP and the commercial vaccine. Although HI and VN titers against both H5 and H7 induced by the VLP vaccine were significantly lower relative to the commercial vaccine, the antibody titers were sufficient to provide good protection, comparable to that of the commercial vaccine (Figure 4). In addition, the VLP vaccine appeared to be more efficient in suppressing lung pathology caused by H7N9 virus at Day 2 p.c., which may be associated with higher levels of HA-binding antibodies against H7 elicited by the bivalent VLP (Figure 3). These findings agreed with previous studies showing that in addition to HI and VN antibodies, total HA-binding antibodies induced by H7 N9 vaccines are important to protection (51, 67–69). The VLP vaccine technology could allow prompt update for the emergence of variant viruses. Currently, HPAI H5N1, and H7N9 viruses undergo continuous antigenic variation which may result in vaccine failure. Because the VLP is produced in a cell-based system, the methodology may be more efficient than the egg-based system in rapid production of vaccines during epidemics of newly emerging viruses. In addition, the VLP strategy has potential advantages over the traditional egg-based inactivated influenza vaccines owing to its capacity for rapid vaccine production based on regional HPAI strains with the unnecessity of using high biocontainment facilities. Furthermore, compared with the commercial whole-virus inactivated vaccines, an additional benefit of the VLP vaccines is the capacity of differentiating infected from vaccinated animals (DIVA) since VLP vaccines do not induce antibodies to the viral internal proteins, such as the NP protein (26, 30). Lastly, single vaccination of chickens with the bivalent VLP elicited immunogenicity and conferred clinical protection comparable to that elicited by booster with other VLP vaccination (32, 36, 39, 45). However, for ultimately being proposed as a critical alternative to the traditional ovoculture vaccines in poultry, some challenges still need to be addressed for the VLP vaccine, mainly including optimization of the manufacturing processes to achieve higher antigen yields, minimization of the downstream processing cost of vaccine manufacturing as well as enhancements of broad protection against multiple variants and subtypes of influenza virus. Last, but not least, it is essential to establish the whole VLP production pipeline, from the upstream cell fermentation and antigen production to the downstream antigen quality-control and process, in vaccine enterprises for large-scale industrial production (58) and application of VLP vaccines in the field.

In conclusion, a non-egg-based bivalent H5+H7 VLP vaccine candidate was generated in this study. A single dose of this vaccine induced efficient serum antibody responses and conferred good protection against homologous virus challenge in chickens. Furthermore, viral shedding and viral replication was significantly inhibited in bivalent VLP-vaccinated birds after challenge with H5N1 and H7N9 viruses, which was comparable to that for the commercial inactivated vaccine. Notably, the VLP vaccine showed advantage in suppressing the lung injury caused by H7N9 virus infection. Therefore, the bivalent H5+H7 VLP vaccine generated in this study can serve as a critical alternative for the traditional egg-based inactivated vaccines to mitigate H5N1 and H7N9 infections in chickens and consequently protect public health. In addition, our study also presented new information for generation of bivalent influenza VLP vaccines for poultry.

## Data Availability Statement

The original contributions presented in the study are included in the article/supplementary material, further inquiries can be directed to the corresponding author/s.

## Ethics Statement

The animal study was reviewed and approved by 1. Guide for the Care and Use of Laboratory Animals of the Ministry of Science and Technology of the People's Republic of China; 2. Jiangsu Administrative Committee for Laboratory Animals (approval number: SYXK-SU-2016-0020), and complied with the guidelines of Jiangsu Laboratory Animal Welfare and Ethics of Jiangsu Administrative Committee of Laboratory Animals.

## Author Contributions

XL and JH conceived and designed the experiments, analyzed the data, and wrote the manuscript. JH, PP, JL, QZ, and RL performed the experiments and analyzed the data. XW, MG, ZH, SH, XL, XJ, and DP provided good suggestions for the experiments and helped to analyze the data. All authors contributed to the article and approved the submitted version.

## Funding

This work was supported by Jiangsu Province Agricultural Science and Technology Independent Innovation Funds [CX (21) 3141], the National Natural Science Foundation of China (32072832), the Postgraduate Research and Practice Innovation Program of Jiangsu Province (KYCX21_3277), the Earmarked Fund for China Agriculture Research System (CARS-40), and a project funded by the Priority Academic Program Development of Jiangsu Higher Education Institutions.

## Conflict of Interest

The authors declare that the research was conducted in the absence of any commercial or financial relationships that could be construed as a potential conflict of interest.

## Publisher's Note

All claims expressed in this article are solely those of the authors and do not necessarily represent those of their affiliated organizations, or those of the publisher, the editors and the reviewers. Any product that may be evaluated in this article, or claim that may be made by its manufacturer, is not guaranteed or endorsed by the publisher.
